# 2740. Letermovir for the Prevention and Treatment of Cytomegalovirus Infection in Solid Organ Transplant Recipients

**DOI:** 10.1093/ofid/ofad500.2351

**Published:** 2023-11-27

**Authors:** Xhilda Xhemali, Courtney A Urzen, Kristen A Blackmer

**Affiliations:** Cleveland Clinic, Cleveland, Ohio; Cleveland Clinic, Cleveland, Ohio; Cleveland Clinic, Cleveland, Ohio

## Abstract

**Background:**

Cytomegalovirus (CMV) is a common infection that can lead to end-organ damage and graft rejection in solid organ transplant (SOT) recipients. Due to the increase in drug-resistant CMV and unfavorable side effect profile of available CMV-active agents, there is a need for additional prophylactic and treatment strategies in this patient population. The goal of this study is to evaluate SOT recipients prescribed letermovir for prophylaxis and/or treatment of CMV.

**Methods:**

This study was a retrospective review of adult SOT recipients in the Cleveland Clinic Health System prescribed letermovir from 11/8/2017 to 8/1/2022. The primary objective was to describe the utilization of letermovir in SOT recipients. Secondary objectives included: proportion of SOT recipients that experienced CMV breakthrough on letermovir prophylaxis, proportion of SOT recipients that experienced refractory CMV infection on letermovir treatment, prevalence of letermovir resistance, and letermovir-induced adverse effects.

**Results:**

A total of 45 patients were included in the study, with 52 letermovir courses. Lung was the most commonly transplanted organ (35%), followed by kidney (22%). 78% of patients were high-risk CMV and the most common immunosuppression regimen consisted of a calcineurin inhibitor in combination with a corticosteroid (82%). 11 patients received letermovir for primary CMV prophylaxis, with only one patient experiencing new/worsening viremia while on the agent. Of the 34 patients who received letermovir for secondary CMV prophylaxis, 11 experienced new/worsening viremia. 7 patients received letermovir for CMV treatment, at a median viral load of 991 IU/mL (IQR 603-2,738) on initiation and peak viral load of 3,892 IU/mL (IQR 1,407-10,209) while on therapy. No patients in the treatment group experienced refractory infection. 7/52 (13.5%) letermovir courses were discontinued due to new/worsening viremia, with no letermovir resistance identified. No letermovir-related adverse effects were documented.

**Conclusion:**

Our study shows that letermovir appears to be effective in suppressing CMV viremia. Letermovir may be an alternative option for SOT recipients who are unable to tolerate or have resistance to traditional CMV therapeutics.

**Disclosures:**

**All Authors**: No reported disclosures

